# Comparative Evaluation of Continuous Lumbar Paravertebral Versus Continuous Epidural Block for Post-Operative Pain Relief in Hip Surgeries

**DOI:** 10.5812/kowsar.22287523.3348

**Published:** 2012-01-01

**Authors:** Pankaj N Surange, Brig Chadalavada Venkata Rama Mohan

**Affiliations:** 1 Interventional Pain and Spine Centre, New Delhi, India; 2 Armed Forces Medical College, Pune, India

**Keywords:** Lumbar Vertebrae, Pain, Postoperative, Hip

## Abstract

**Background:**

Effective control of postoperative pain remains one of the most important and pressing issues in the field of surgery and has a significant impact on our health care system. In too many patients, pain is treated inadequately, causing them needless suffering and they can develop complications as an indirect consequence of pain. Analgesic modalities, if properly applied, can prevent or at least minimize this needless suffering and these complications.

**Objectives:**

The aim of this study was to compare the efficacy of continuous infusions of local anesthetic drugs by paravertebral and epidural routes in controlling postoperative pain in patients undergoing hip surgeries.

**Patients and Methods:**

The study involved 60 patients who were undergoing hip surgery under the subarachnoid block. They were randomly divided into 2 groups of 30 patients. Group I (paravertebral group) received a single dose of spinal anesthesia with 2.5 mL 0.5% bupivacaine (heavy) + a continuous infusion of 0.125% bupivacaine at 5 mL/h in the paravertebral space. Group II (epidural group) received a single dose of spinal anesthesia with 0.5% bupivacaine (heavy) + a continuous infusion of 0.125% bupivacaine at a rate of 5 mL/hr in the epidural space for 48 hours in the postoperative period. Visual analogue scale (VAS) score, vital statistics, rescue analgesia, and procedure time were compared with the corresponding times between the 2 groups by student’s t-test and repeated measures ANOVA with post hoc Bonferroni. P < 0.05 was considered significant. There were no statistically significant differences between the 2 groups regarding mean pain score in the first 48 hours.

**Results:**

Mean arterial pressure was significantly lower in the epidural group compared with the paravertebral group from 2 hours after start of the infusion until 48 hrs. Regional anesthesia procedure time was significantly longer in the epidural group (P < 0.001). There was no significant difference between the 2 groups regarding frequency of postoperative complications and catheter-related problems.

**Conclusions:**

The results of our study indicate that for patients who are scheduled for hip surgery, both continuous paravertebral and continuous epidural analgesia are effective in controlling postoperative pain but that the former has several crucial advantages.

## 1. Background

The conquest of pain that is inherent to surgery is the most important event in the history of mankind. Pain following surgery is a universal phenomenon; yet, it is often underestimated and undertreated. This is likely due to difficulties in balancing an effective postoperative pain treatment regimen with acceptable side effects.

Effective control of postoperative pain remains one of the most important and pressing issues in the field of surgery and has a significant impact on our health care system. Most of the hundreds of millions of people worldwide who undergo operations each year experience pain of varying intensity. In too many patients, pain is treated inadequately, causing them needless suffering, and they can develop complications as an indirect consequence of pain. Analgesic modalities, if properly applied, can prevent or at least minimize this needless suffering and these complications.

Pain following hip surgery, which constitutes the majority of operations (1), especially in elderly with other comorbid conditions, is particularly severe, as this major joint operation entails massive nociceptive inputs from richly innervated joint tissue that produces continuous deep somatic pain and bouts of severe reflex spasm of the muscle that is supplied by the same and adjacent spinal cord segment and are superimposed onto the incision pain. The incidence, intensity (severity), and duration following hip surgery hare estimated to be moderate in 30% to 40% and severe in 40% to 50%, and the duration ranges from 2-4 days (2).

Adequate postoperative pain relief improves the surgical outcome in terms of reduced morbidity, reduced hospital stay in the postoperative period (3), and reduced postoperative organ dysfunction (4) due to lower surgical stress. Various pharmacological and nonpharmacological methods have been used to provide postoperative pain relief. Such agents include oral analgesics, intramuscular (IM) or intravenous (IV) narcotics, nonsteroidal anti-inflammatory drugs, subanesthetic doses of ketamine, sublingual buprenorphine hydrochloride, intrathecal and epidural drugs, non-narcotics like clonidine, midazolam, ketamine, intra pleural local anesthetics or opiods, local anesthetic infiltration at the line of incision, inhalation agents (such as nitrous oxide), cryoanalgesia, transcutaneous nerve stimulation, and acupuncture. However, they effect varying results.

Some of these techniques are also associated with unpredictable responses and complications. When compared with other techniques, many regional analgesic techniques, such as epidural and paravertebral blocks, provide superior pain relief, may favorably influence outcomes with regard to blood loss and thromboembolic events (5), and can lead to substantial reductions in surgical stress responses (6).

## 2. Objectives

The purpose of this study was to compare the efficacy of postoperative pain relief after hip surgery using lumbar epidural and lumbar paravertebral anesthetic (Psoas compartment block) with a continuous infusion of 0.125% bupivacaine.

## 3. Patients and Methods

A randomized, controlled comparative study of 60 patients who were scheduled for hip surgery was performed. After the ethical committee approved the study design, we enrolled American society of anesthesiologist (ASA) class I, II, and III patients who were scheduled for unilateral hip surgery. Written informed consent was obtained.

Patients with the following criteria were excluded from the study:

1. Lack of patient consent
2. Sepsis over lumbar vertebra
3. Patient on chronic analgesic/anticoagulant therapy
4. Patient with a neurological disorder
5. Known allergy to any local anesthetic
6. Dementia that prevented proper comprehension

Sixty such patients formed the total sample size and were randomly allocated into groups of 30 subjects.

1. Group I: 5 mL/h, 0.125% bupivacaine, continuous paravertebral group
2. Group II: 5 mL /h, 0.125% bupivacaine, continuous epidural group

Informed written consent was obtained from each patient after the study protocol and use of the visual analog scale were explained and after we gained their confidence during the preanesthetic assessment visit on the night before surgery.

### 3.1. Preanesthetic Assessment

During the preanesthetic assessment, a detailed history of each patient was taken and recorded in Performa. A thorough clinical examination was performed. Detected to be having any of the conditions mentioned in exclusion criteria were excluded from the study. Hematological measures, such as hemoglobin, blood count, bleeding time, and clotting time, were recorded, and routine and microscopic urine analysis was performed. Chest X-ray and electrocardiogram (ECG) were taken whenever necessary. All patients were visited the night before the surgery and had the procedure explained to them; after being advised and reassured, their confidence was gained. All patients were advised to fast after 2200 hours the night before the surgery.

### 3.2. Preoperative Medication

All patients were given tablet alprazolam 0.25 mg orally at 10 pm in the night before surgery.

### 3.3. Technique

Patients pulse rate, ECG, and noninvasive blood pressure were recorded, and a wide-bore intravenous line was established. Patients were preloaded with Ringer’s solution at 15 mL/kg body weight 30-60 minutes before intrathecal drug administration. Patients were positioned in the sitting position, supported, and chin-flexed on the chest; those who were unable to sit were positioned in the lateral decubitus position. The back was prepared using Ioprep and wiped with methylated spirit, and the area was draped with a sterile towel.

#### 3.3.1. Group I

To locate the puncture site, a point was made on the upper border of the spinous process of the L2 vertebra, and we identified the punctured site 3 cm lateral to the first point on the target side. Local anesthetic was infiltrated at the puncture site, and a 16-G Tuohy needle was advanced perpendicular to all planes until it made contact with the transverse process of the L2 vertebra.

It was then redirected slightly caudal to the transverse process and advanced 1-2 cm to reach the Psoas compartment. The stylet was removed, and 10 mL of saline was injected to expand the compartment. Next, an 18-G catheter was inserted through the Tuohy needle and advanced approximately 4 cm caudally within the compartment. Initially, a 3-mL test dose solution containing 2% lidocaine and 1:200,00 epinephrine was injected via the paravertebral catheter.

Then, a lumbar puncture was performed through the L3-L4 interspace, and 2.5 to 3.0 mL of 0.5% bupivacaine was injected.

#### 3.3.2. Group II

The L3-L4 interspace was identified and infiltrated with local anesthetic. A 16-G Tuohy needle was inserted through the the L3-L4 interspace, and the epidural space was located by loss of resistance technique. The stylet was removed, and a 3-mL test dose solution containing 2% lidocaine and 1:200,00 epinephrine was injected. Then, a lumbar puncture was performed with a 25-G spinal needle, which was passed through the Tuohy needle, and 2.5-3.0 mL of 0.5% bupivacaine was injected. The catheter was then advanced approximately 2-3 cm cephalad and secured.

### 3.4. Monitoring

The cephalad spread of analgesia and the degree of motor blockade of the lower limb were recorded. The level of sensory blockade was assessed using a 25-gauze short-bevel needle and recorded as analgesia to loss of sensation to a pin prick (4). The following parameters were monitored continuously in all patients and recorded.

1. Heart rate and ECG
2. Noninvasive blood pressure
3. SPO2 (pulse oximetry)
4. Blood loss
5. Urine output
6. IV fluid input

Patients were observed for any discomfort, nausea, vomiting, shivering, pain, bradycardia, and any other side effects, and the need for any additional medications was recorded. IV fluids were administered in the form of Ringer’s lactate, dextrose normal saline, and colloids in calculated doses, depending on the weight of the patient, and further adjusted as per blood loss during surgery. Hypotension of more than 30% of the preanesthetic value was treated with rapid infusion of fluids and an injection of mephenterine intravenously. Bradycardia (heart rate less than 60/mt) was treated with intravenous atropine sulphate.

### 3.5. Postoperative Observation

At the end of the operation, each patient was connected to an infuser set to deliver an infusion of 0.125% bupivacaine at a rate of 5 mL per hour for 48 hours. All patients were observed in the postanesthesia care unit and then in the ward. Immediately after surgery, each patient started identical physical therapy regimens. From postoperative Day 1, patients performed active and assisted hip flexion and extension exercises against gravity twice daily. Getting up from bed was encouraged as soon as possible, followed by ambulation with a walker. Several parameters were assessed postoperatively.

1. Regional anesthesia procedure time; i.e., the period from positioning the patient for technique to catheter fixation.
2. Operation time
3. Severity of pain using the 10-cm visual analog scale and vital parameters, such as pulse, mean arterial pressure (MAP), and respiratory rate (RR) at 2, 4, 8, 12, 18, 24, 32, 40, and 48 hours
4. Supplemental analgesic requirement
5. Catheter-related problems and any complications in first 48 postoperative hours
6. Rescue analgesia was provided by Inj. diclofenac 1 mg/kg IM

## 4. Results

A total of 60 patients were studied. They were randomly divided into 2 groups of 30 patients each. Six patients were excluded from the study. The data that we present are from the remaining 54 patients (Table 1). 

**Table 1.** Patients’ Charecterestics

	Group I, (n = 28)	Group II, (n = 26)
Age ^a^, y, Mean ± SD	70.26 ± 10.25	65.8 ± 12.65
Sex, No. ^b^		
Male	16	16
Female	12	10
Weight^c^, kg, Mean ± SD	63.96 ± 9.04	63.40 ± 7.64
Height ^d^^,^^e^, cm, Mean ± SD	155.47 ± 2.54	156.27 ± 3.07
ASA Status ^f^		
ASA-I	9	9
ASA-II	14	13
ASA-III	5	4
Type of Surgery, No.		
Hemiarthroplasty ^g^	14	12
ORIF ^k^ with DHS ^g^^, ^^k^	11	10
THR^g^^,^^k^	2	3
External fixator ^g^	1	1
Duration of surgery ^g^, Mean ± SD	92.83 ± 15.1	94.5 ± 16.93
Regional anesthesia procedure		
Time, min ^h^, Mean ± SD	10.633 ± 1.29	15.66 ± 1.52
Attempts, Mean ± SD	1.3 ± 0.5	1.6 ± 0.9
Injection of diclofenac sodium^i^, No.	2.88	2.66
Side effects ^j^^,^^d^, No.		
Nausea and vomiting	2	3
Hypotension	0	2
Dislocated catheter	2	1

^a^ Analysis of the data showed no significant statistical difference between them.

^b^ Analysis of the data showed no significant statistical difference between them.

^c^ Comparison of mean body weight of the two groups showed no Significant.

^d^ P value is not significant (P > 0.05).

^e^ The mean body height between two groups showed no significant statistical difference.

^f^ Patients of ASA-I, ASA -II and ASA -III are included in the study and their distribution is given.

^g^ P value is not significant (P > 0.05).

^h^ Procedure time was significantly longer in epidural group (P < 0.001).

^i^ Rescue analgesia in 48 hours.

^j^ There was no significant difference between the two groups regarding frequency of postoperative complication and catheter related problems.

^k^ Abbreviations: DHS, dynamic hip screw fixation; ORIF, open reduction and internal fixation; THR, total hip replacement

1. Group I (paravertebral group)- 28 patients
2. Group II (epidural group)- 26 patients 5

### 4.1. Hemodynamic Changes

The hemodynamic parameters that we measured were heart rate and mean arterial pressure. The preoperative heart rate (HR) and MAP of all patients were recorded before the procedure was performed. Subsequent readings were taken every 5 minutes. Intraoperatively after spinal anesthesia and thereafter recorded at 2, 4, 8, 12, 18, 24, 32, 40 and 48 hours.

#### 4.1.1. Mean Arterial Pressure

**Figure 1.**
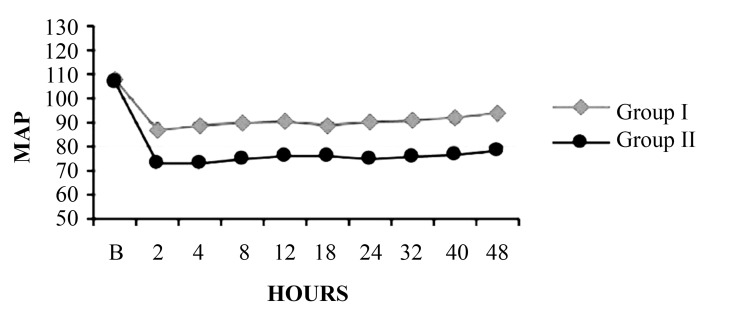
Comparison of Mean Arterial Pressure Between Group I and Group II

Figure 1 shows the mean MAP in both groups. There mean arterial pressure was significantly lower in he epidural group compared with the paravertebral group from 2 hours after the infusion was begun to 48 hours.

#### 4.1.2. Heart Rate

**Figure 2.**
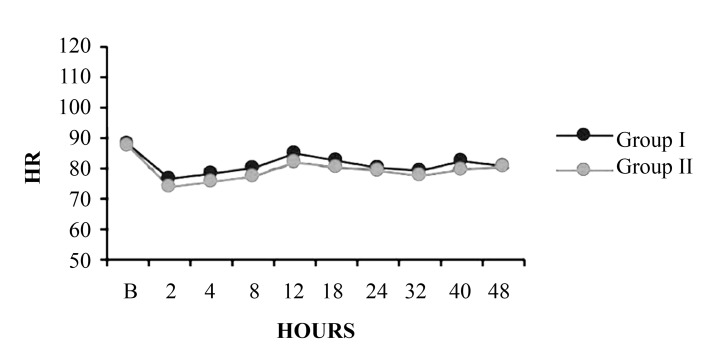
Comparison of Mean Heart Rate Between Group I and Group II

Figure 2 shows the mean HR in each group. The mean heart rate was slightly higher in the epidural group throughout the study period, albeit insignificantly.

#### 4.1.3. Visual Analogue Scale

**Figure 3.**
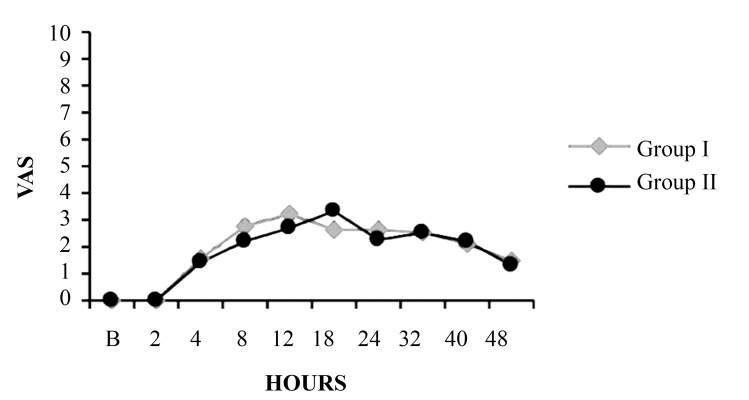
Comparison of Visual Analogue Scale Between Group I and Group II

Figure 3 shows mean visual analogue scale (VAS) in each group. There were no statistically significant differences between the groups with regard to mean pain scores in the first 48 hours.

## 5. Discussion

The results of our study indicate that for patients who are scheduled for hip surgery, both continuous paravertebral and continuous epidural analgesia are effective in controlling postoperative pain but that the former has several crucial advantages. We modified the posterior approach technique of Deckrey et al., Chayen et al. (7), and Winnie et al. (8) slightly, as follows. To locate the puncture site, a point was made on the upper border of the spinous process of the L2 vertebra and identified the punctured site 3 cm lateral to the first point on the target side.

Local anesthetic was infiltrated at the puncture site, and a 16-G Tuohy needle was advanced perpendicular to all planes until it made contact with the transverse process of the L2 vertebra. It was then redirected slightly caudal to the transverse process and advanced 1-2 cm to reach the Psoas compartment. The stylet was removed, and 10 mL saline was injected to expand the compartment. Next, the 18-G catheter was inserted through the Tuohy needle and advanced approximately 4 cm caudally within the compartment to reach the vicinity of the L4 vertebra, based on the finding that most branches of the lumbar plexus are in close proximity in the region of the 4th lumbar vertebra in the Psoas compartment. The efficacy of this postoperative pain management was assessed using the VAS.

Six patients were excluded from the study. In 2 patients, the epidural catheter could not be sited; both patients were aged above 70 years and had mild scoliosis. Three patients-2 in the paravertebral group and 1 in the epidural group—were excluded due to accidental removal of the catheter while transferring them to the ward. One patient from the epidural group withdrew due to persistent hypotension.

Vascular puncture was encountered during the procedure in 2 patients; the needle was redirected cephalad to the transverse process, and the block was administered. The data are from the remaining 54 patients.

There was no significant difference in mean age, sex, height, weight, or ASA class distribution between patients in this study. The various surgeries that were performed and the duration of surgery were comparable between them. We measured the VAS at 2, 4, 8, 12, 18, 24, 32, 40, and 48 postoperatively. We found that both routes were effective in controlling postoperative pain and did not differ significantly, reflecting good postoperative pain control in both groups. The results of our study are consistent with those of of P.J. Mathews (9), who did not observe any significant difference in analgesia by VAS score over 24 hours.

Perttunen et al. (10) demonstrated good postoperative pain relief at rest in paravertebral and extradural groups 1 hour after surgery and found comparable segmental analgesia in both groups up to 20 hours. The rescue analgesia was provided with Inj. voveran 1 mg/kg IM, and there was no significant difference in the number of doses that was required in both groups. However, the first analgesic requirement was earlier in the paravertebral group between 8-10 hours versus 8-12 hours in the epidural group. Intraoperative heart rate, mean arterial pressure, and SPO2 were monitored and were similar in both groups (data not reported). The mean arterial pressure at 2, 4, 8, 12, 18, 24, 32, 40, and 48 hours after starting the continuous infusion with 0.125% bupivacaine at 5 mL/h fell significantly between two groups.

The paravertebral block effects a predominantly unilateral sympathetic blockade, whereas an epidural block is usually bilateral; the extent of the spread of the drugs is also greater. These differences might explain the disparities in the incidence of hypotension between the 2 groups. This conquer with the study G Turker et al. (11), White and Chappell (12), Richardson et al. (13). The mean heart rate, although not statistically significant, was slightly higher in the epidural group throughout the period. This finding may be explained by the hypotension in the epidural group. The regional anesthesia procedure time (i.e., time from positioning the patient to fixation of the catheter) was significantly longer in the epidural group. Our data also showed that the epidural block required more attempts than the paravertebral block. These results might be attributed to positioning difficulties, as the epidural block requires a strict midline position, and to spinal calcification and degeneration, which are frequently encountered in elderly patients.

In our study, both continuous paravertebral and epidural analgesia provides effective postoperative pain relief after hip surgery, but the paravertebral block is technically simple and easy to learn with few contraindications, provides hemodynamic stability, and has a low complication rate and is therefore a safe and effective technique in controlling postoperative pain after unilateral hip surgery.
